# Physicochemical and adulteration study of fresh milk collected from different locations in Pakistan

**DOI:** 10.1016/j.sjbs.2022.103449

**Published:** 2022-09-17

**Authors:** Taufiq Nawaz, Zia Ur Rehman, Rafi Ullah, Nazeer Ahmed, Samy Mahmoud Sayed

**Affiliations:** a Department of Food Science and Technology, The University of Agriculture Peshawar, Khyber Pakhtunkhwa, Pakistan; b Department of Agriculture, University of Swabi, Anbar-23561, Swabi, Khyber Pakhtunkhwa, Pakistan; c Department of Economic Entomology and Pesticides, Faculty of Agriculture, Cairo University, Giza 12613, Egypt

**Keywords:** Adulteration, Composition, Milk, Mardan, Analysis

## Abstract

The present research was carried out to assess raw milk's quality as collected from the commercial markets of the Mardan district (Khyber Pakhtunkhwa). The locality from which milk samples were collected included; Bijligar (BG), Manga (M), Chamthara (CM), Main Bazar Mardan (MB), Mahidherai (MD), and Sharif Abad (SB), located in district Mardan, Khyber Pakhtunkhwa (KP). A total of 36 milk samples were collected at the rate of 6 samples per location. The outcome of the data exhibited that the percentage of protein content was highest (3.34%) in MB and SA (3.30%), while lower percentages were recorded in sample M (3.03%) and CM 93.06%). Maximum pH were shown in M and BG to be 7.55 and 7.33, respectively. For fats content, the highest percentage of fats was witnessed in MB as 4.04%, and minimum fats content was noted in Sample M as 3.57%. Water content was highest in Sample SA and BG at 15.85% and 15.64%, respectively. Qualitative analysis of adulterants like detergents, Formalin, starch, and Hydrogen peroxide was also carried out for all the collected samples. Adulteration results were positive for all the milk samples, with the highest being in samples MB (30%), while all the remaining samples had adulteration at 20% each. Both MB and CM samples were adulterated with urea, while the remaining 4 were adulterated with neutralizers. Thus, it may be summarized from the whole analysis that the milk available in commercial markets of district Mardan was adulterated with different adulterants and is not recommended for consumption.

## 1 Introduction

The livestock division shows an energetic character in the economy of Pakistan and shares a GDP of 11.1% globally, and takes 5th position in the milk-producing country worldwide. Livestock is the main source for poor people to earn money to live their lives (Ahmad, 2018). It was estimated that about 30–35 million people were connected to livestock and labor. In Pakistan, Nili- Ravi Buffaloes and Sahiwal breed gives good milk and produces higher income. Currently, milk-productive animals shared buffaloes (32.7), sheep (28.4), cows (36.9), goats (63.1), and camels (1.0) produce 47.95 million tonnes of milk. The accessibility of milk per person is around 218 L as compared to the world's best countries like Finland yields183.9 L, Australia yields 106.3 L, and the US yields 83.9 L, according to an economic survey 2012 (Anonymous, 2012). The structure of buffaloes and cow milk displays the collection of fats (7.6, 4.5%), lactose (5.1, 4.9%), ash (0.78, 0.72%), protein (3.8, 3.8%), and complete solids (17.0, 13.9%), respectively (Khan et al., 2005, Farooque, 2017).

The major problems these days are milk adulteration and a serious threat to the milk industry of our country. It is also very harmful to humans (Barham et al., 2018). Poor people try to contaminate food by various adulteration techniques like oil of plant bases such as flour, sugarcane, skim milk powder, whey powder, vegetables, starch, flour, and other elements which increase the hard contents of the milk and decrease the liquid contents and thus reduces the quality of milk and increases the quantity of milk. (Fakhar et al., 2006). The visual sense of milk and milk-related products were also increased by adding other harmful substance like detergents. Adding water to milk decreases the solid part and reduces the foamy look of the milk. Hence the use of detergents gives the foamy milk appearance to fraud people. Also, adding other adulterants to milk, like urea and hair removal powder, gives the milk an aesthetic white color. A touch of urea is sufficient to increase the visual sense of milk (Walker et al., 2004).

The common additives used in milk are shampoo, detergents, washing powder, and urea, decreasing milk quality and increasing milk quantity. Nowadays, a chemical (formaldehyde) used as a preservative to preserve meat is used as a milk preserver from spoiling milk for a long time. The structure of formaldehyde is made from oxygen, hydrogen, and carbon. According to the FDA, formaldehyde (cancer-causing agent) is used in food and non-food materials, like preserving human and animal bodies from rotting, and also in cosmetic products (Gadhi, 2019). To increase the milking ability of dairy animals, people used oxytocin injections on animals for up to 20–30%. Pakistan is in 5th position in milk production internationally and produces 28 billion liters. Health professionals determined that the contamination of milk causes serious health problems like hepatitis, failure of kidneys, heart problems, stomach tumors, cancer, nausea, asthma, pneumonia, and allergic reactions (Gadhi, 2019). The impact of milk in our daily routine and various contaminated techniques in milk supply paths should be in mind. The present effort is to investigate “Various methods of Adulteration in Milk in Peshawar. So the study's goals were to determine adulteration in local milk samples and conclude the exact adulterants and their amount. Furthermore, the sample's chemical structure will be examined, as well as the content of proteins, fats, water, etc.

## 2 Materials and methods

During this research, the locality from which milk samples were collected included Bijligar (BG), Manga (M), Chamthara (CM), Main Bazar Mardan (MB), Mahidherai (MD), and Sharif Abad (SB), located in the district Mardan, KP (Table 1). A total of 36 milk samples (250 ml each) were collected. After collecting samples, each sample was sterilized, capped and labeled, and then transferred to a special box containing ice cubes. Without delay, these boxes were then transported to the Food laboratory of the Khyber Pakhtunkhwa (KP) Food Safety & Halal Food Authority in Peshawar. The samples were tagged and labeled for collection date, time of collection, and name of samples according to their location. These were then analyzed for different lab tests in triplicate, and readings were taken. During testing, hygiene and safety protocols were followed to avoid any contamination.

**Table 1:** Fresh milk samples collected from various regions.

S. No.	Treatments IDs	Area from which sample is collected
1	MD	MahiDaray
2	CM	Chamtara
3	SB	Sharifabad
4	M	Mangah
5	BG	Bijligar
6	MB	Mardan main bazzar

### 2.1 Physical examination

Physical examination of all the milk samples were done for different characteristics like settling, color, odor, and consistency according to the methods described by Khan et al. (2005).

### 2.2 Physico-chemical composition

Analysis of such composition was completed for all the samples, which included density, SNF (solid, Not Fats), protein, water (added), lactose, electrical conductivity (EC), and pH. Scan S-60 was used to determine fat content, while SNF and Total solids were calculated with the methods used by Khan et al. (2005) with slight modification.

### 2.3 Physical and chemical analysis

According to Khan et al. (2005), Acidity and specific gravity were determined with required modifications.

### 2.4 Milk adulterants

Different milk adulterants, including urea, added water, starch, hydrogen peroxide, detergents, and Formalin, were determined according to Tipu et al. (2007).

### 2.5 Statistical analysis

All the data noted from this research was statistically analyzed using Originpro 2019. The post-Hoc test was used for one-way ANOVA and LSD for mean separation. Statistical differences were indicated using P ≤ 0.05 (Steel et al., 1997).

## 3 Results

### 3.1 Fat content (%)

**Fig. 1:**
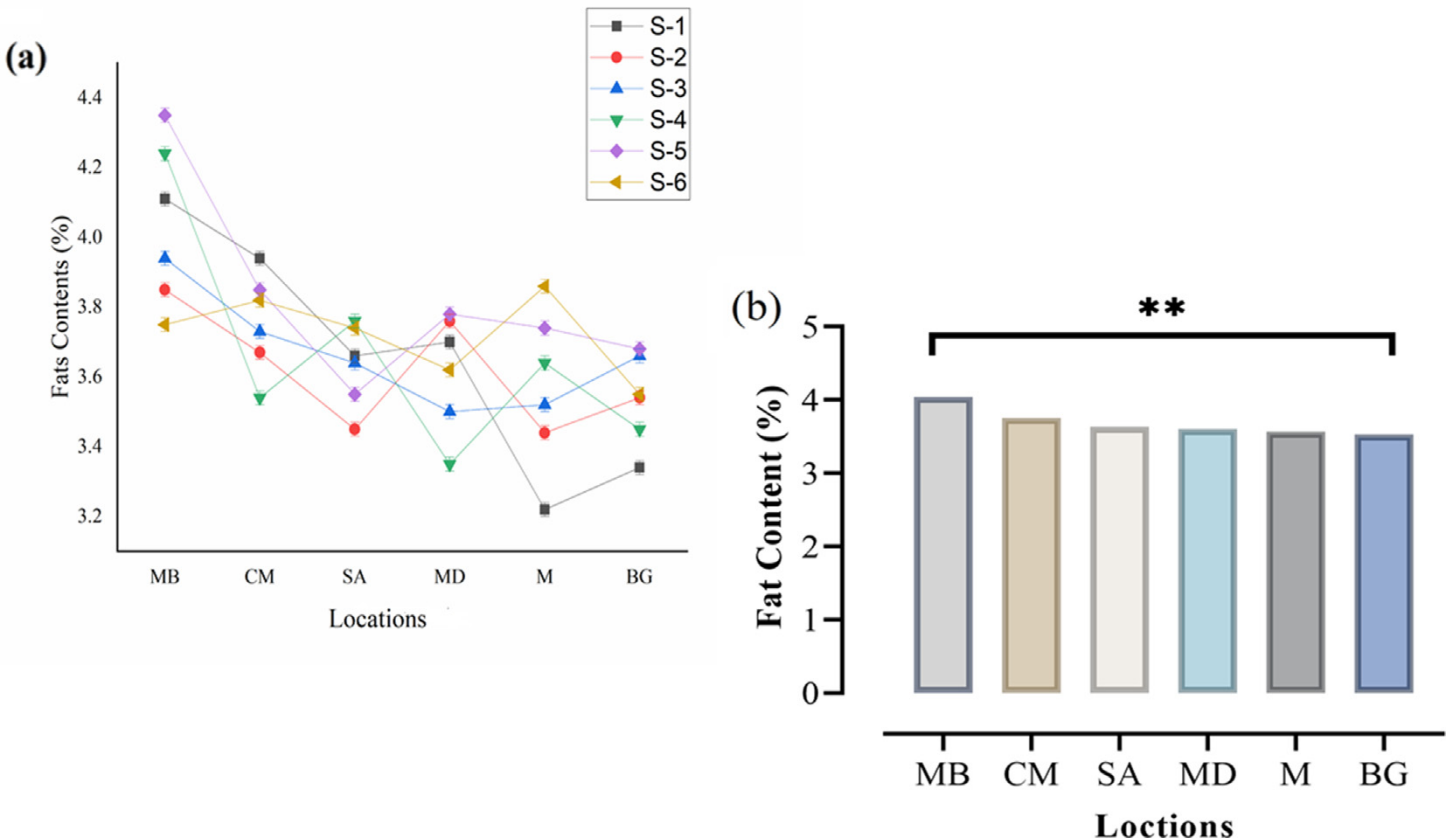
Content of fats present in the fresh milk collected from different regions.

. Sample (MB) showed the highest value of fats at 4.04%, followed by sample (CM) at 3.75%. The minimum value of fat content was noted to be 3.57% and 3.53% in samples M and BG alternatively. Furthermore, the Highest Fat content (%) was recorded on MB and the lowest on BG, which were significantly different from each other (Fig. 1a, b).

### 3.2 Protein content (%)

**Fig. 2:**
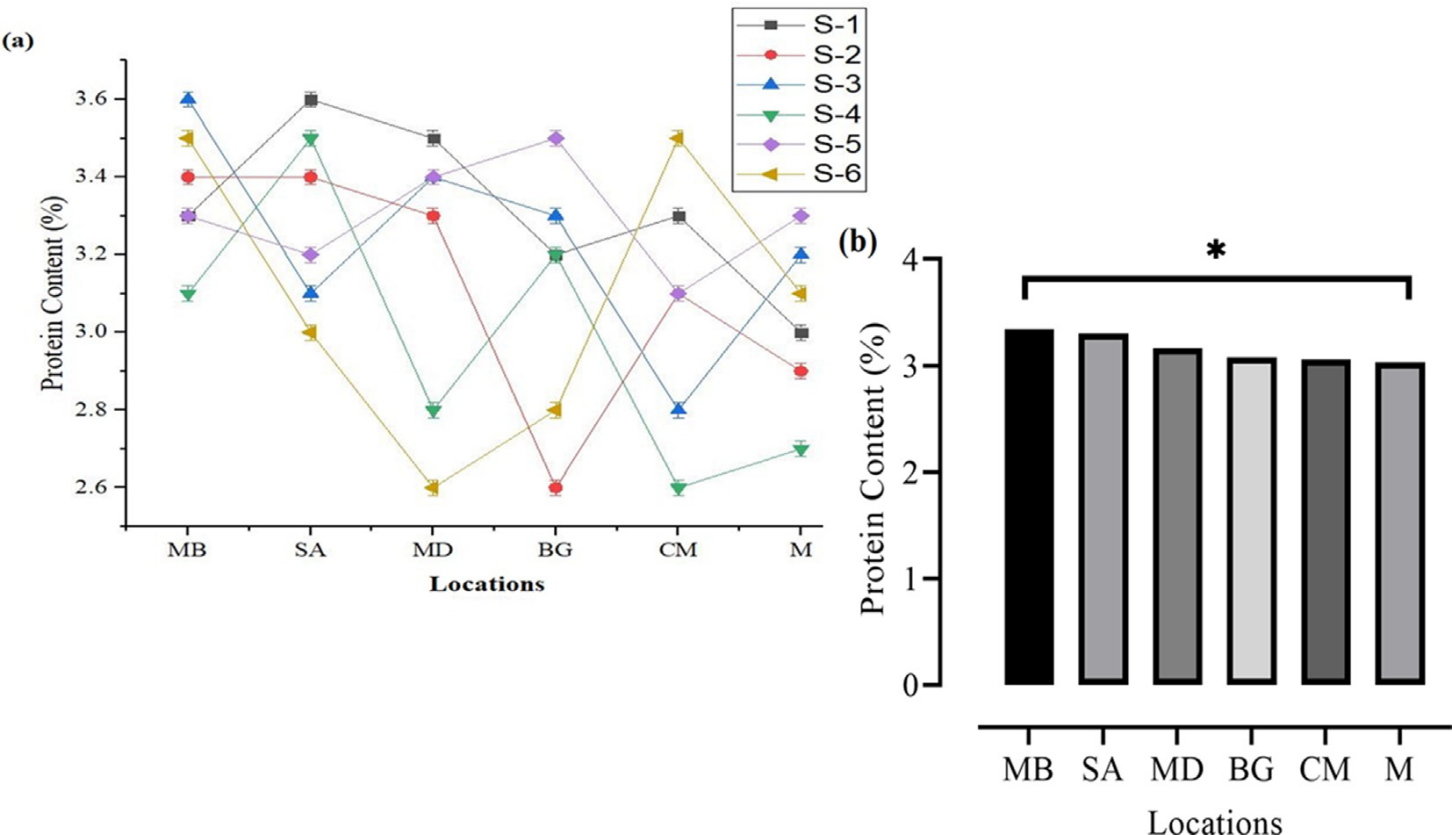
Presence of protein content in the fresh milk collected from different regions.

. Sample (MB) showed the highest protein value at 3.34%, followed by sample (SA) to be 3.30%, and the minimum value of fat content was noted to be 3.03% and 3.06% in samples M and CM alternatively. Similarly, the highest Protein content (%) was recorded on MB, whereas the lowest was observed on M (%) (Fig. 2a, b).

### 3.3 Water content (%)

**Fig. 3:**
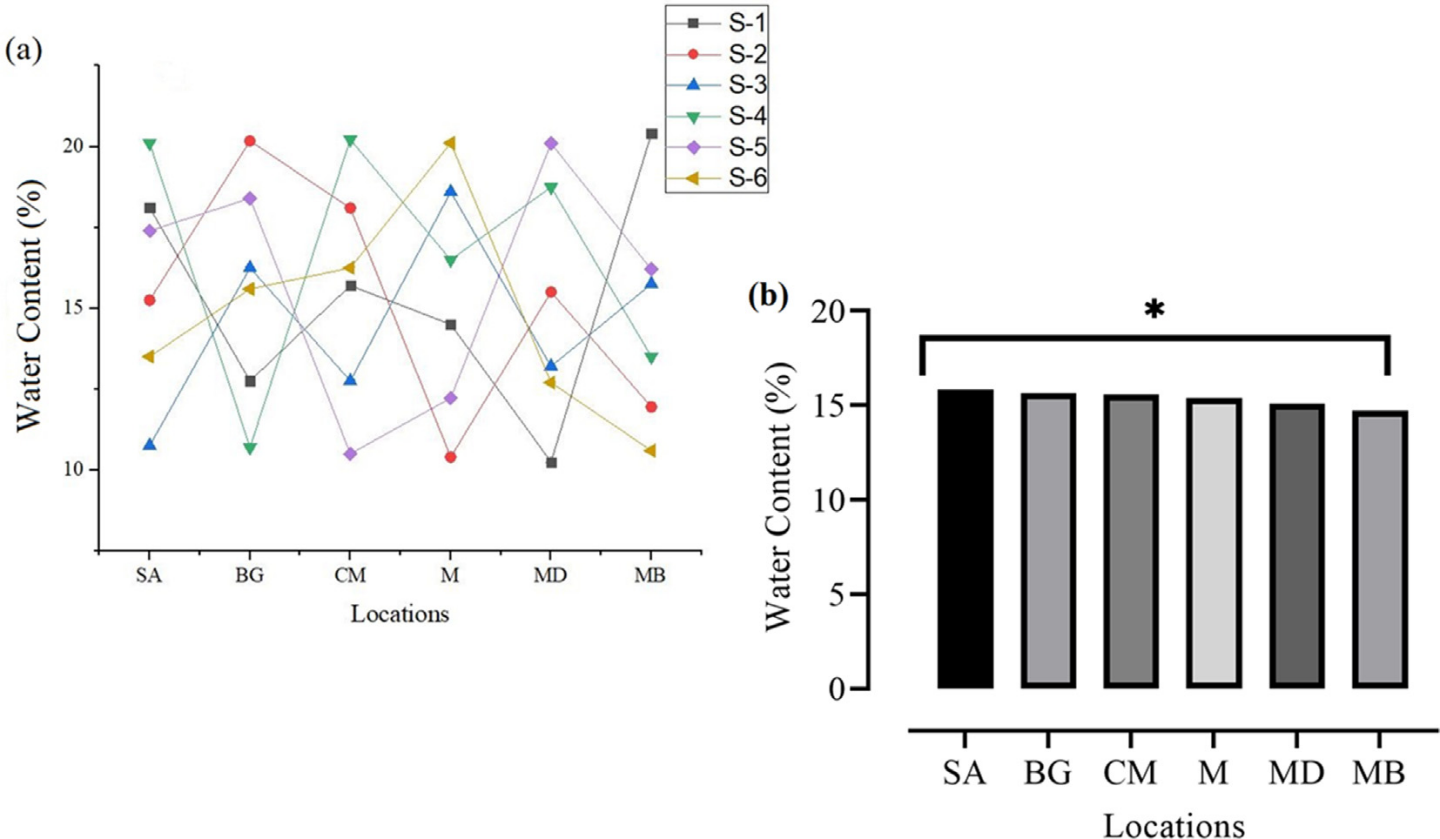
Presence of water content in the fresh milk samples collected from different regions.

Sample (SA) showed the highest water content value at 15.85%, followed by sample (BG) at 15.64%. The minimum water content value was 15.08% and 14.73% in samples MD and MB. Furthermore, the maximum water content (%) was recorded on SA, and the minimum was observed on MB (Fig. 3a, b).

### 3.4 pH

**Fig. 4:**
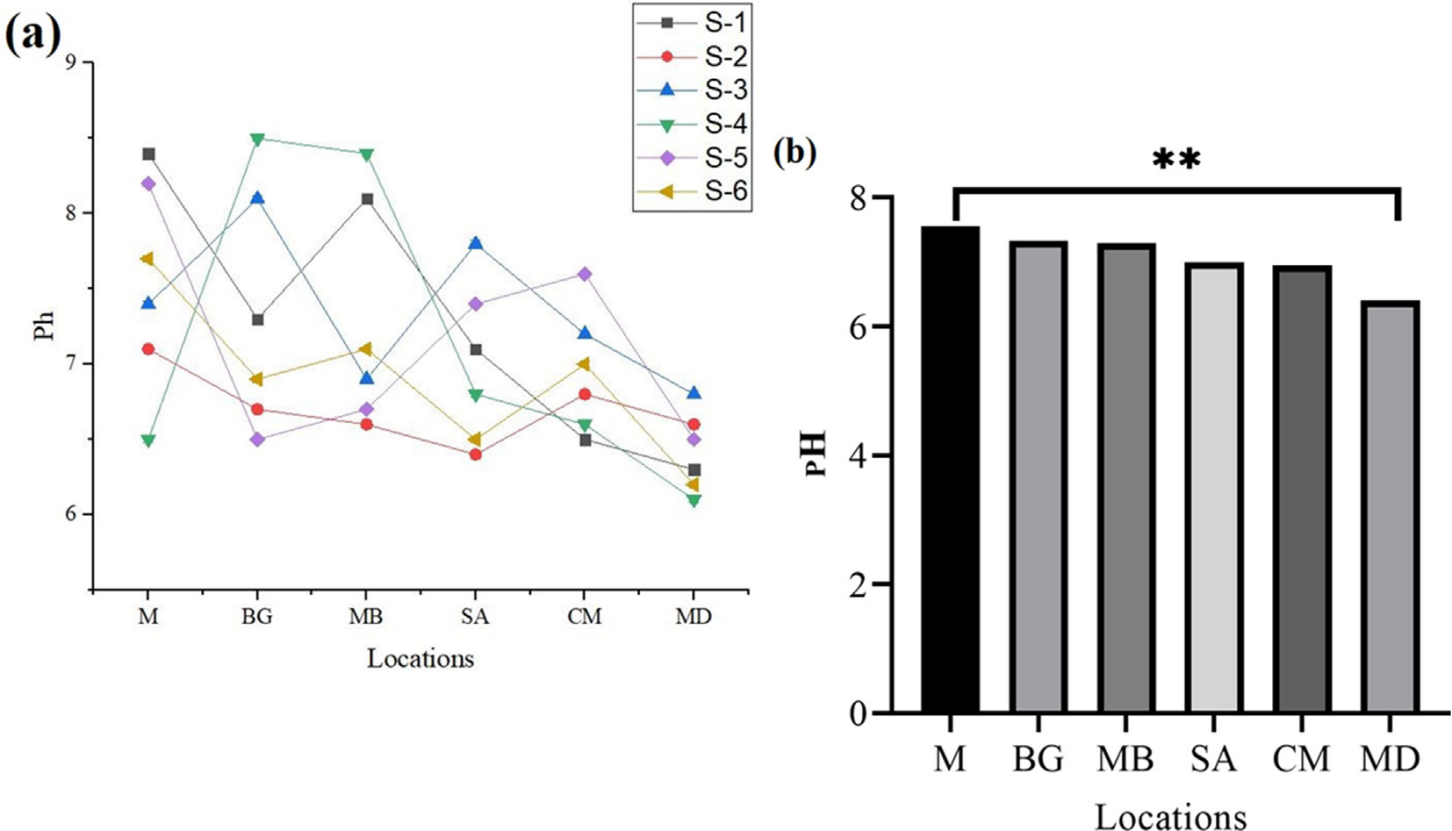
Fresh milk pH collected from different regions.

The results of the experiment regarding pH determination showed mean pH readings to be 7.55 (M), 7.33 (BG), 7.3 (MB), 7.0 (SA), 6.95 (CM), and 6.41 (MD). Top mean values were witnessed in sample M (7.55) and sample BG (7.33), respectively, while sample MD (6.41) and CM (6.95) were at the bottom of mean pH values, respectively. Nevertheless, the highest pH value was observed on M and the lowest in MD (Fig. 4a, b).

### 3.5 Density

**Fig. 5:**
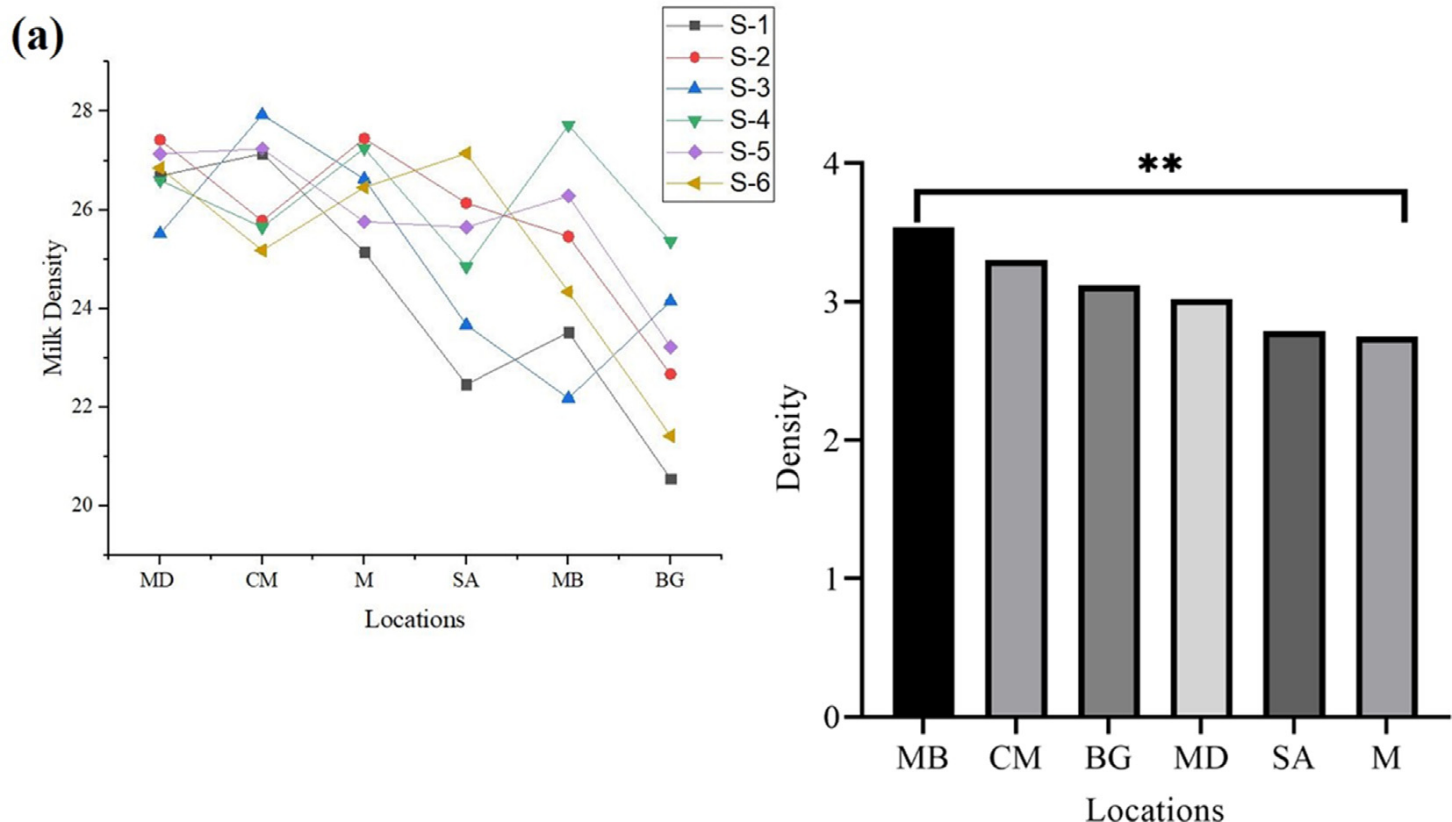
Fresh milk density collected from different regions.

Top mean values were witnessed in the sample MD (26.70) and sample CM (26.48), respectively, while sample BG (22.89) and MB (24.91) were at the bottom of mean density values, respectively. In conclusion, the Highest density was observed in MB and the lowest in M (Fig. 5a, b).

### 3.6 Lactose

**Fig. 6:**
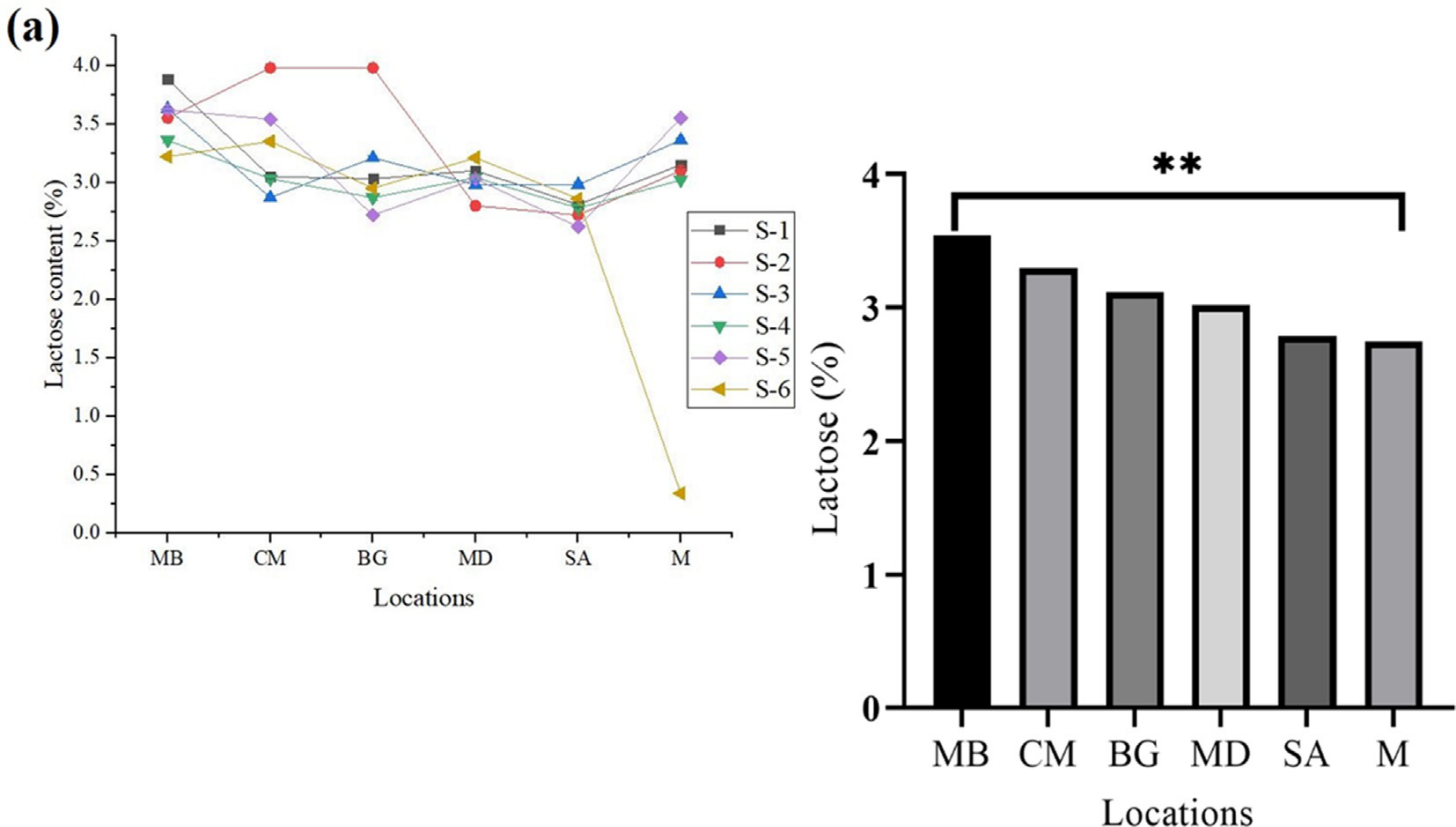
Lactose contents in fresh milk collected from different regions.

The highest mean values were witnessed in sample MB (3.54) and sample CM (3.30), respectively, while sample M (2.75) and M (2.75) had the lowest mean values for lactose content. In conclusion, the highest lactose (%) was observed in MB and the lowest in M (Fig. 6a, b).

### 3.7 Salts

After the determination of salt content, mean values were recorded as 0.46 (MD), 0.49 (CM), 0.46 (SA), 0.56 (M), 0.45 (BG) and 0.53 (MB) alternatively, as shown in Table 2. Sample (M) showed the highest salt content as 0.56, followed by sample (MB) to be 0.53, and the minimum salt content was noted to be 0.45 and 0.46 in BG and MD, alternatively.

**Table 2:** Presence of salts in fresh milk obtained from different regions.

Treatment	S-1	S-2	S-3	S-4	S-5	S-6
**MD**	0.49	0.44	0.47	0.44	0.48	0.45
**CM**	0.42	0.48	0.58	0.52	0.45	0.55
**SA**	0.45	0.42	0.52	0.48	0.42	0.49
**M**	0.5	0.56	0.62	0.65	0.52	0.58
**BG**	0.48	0.42	0.47	0.51	0.36	0.38
**MB**	0.64	0.52	0.45	0.42	0.48	0.5

### 3.8 Conductivity

**Fig. 7:**
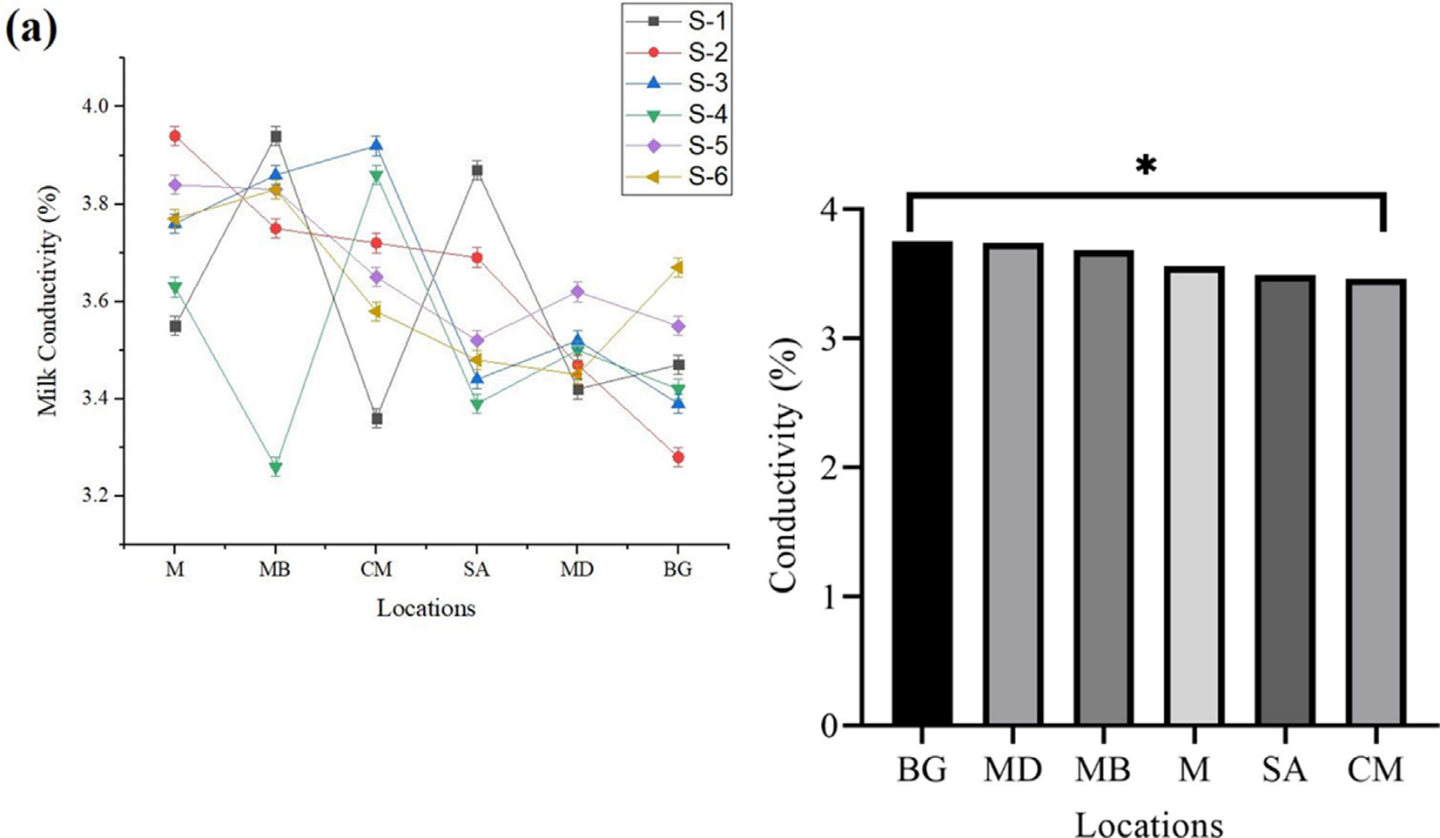
Conductivity of the fresh milk collected from various regions.

Sample (BG) showed the highest conductivity value as 3.75, followed by sample (MD) to be 3.74, and the minimum conductivity value was noted to be 3.46 and 3.49 in sample CM and SA, alternatively. In conclusion, the Highest milk conductivity (%) was observed in BG and the lowest in CM (Fig. 7a, b).

### 3.9 Solid Non-Fat (SNF)

**Fig. 8:**
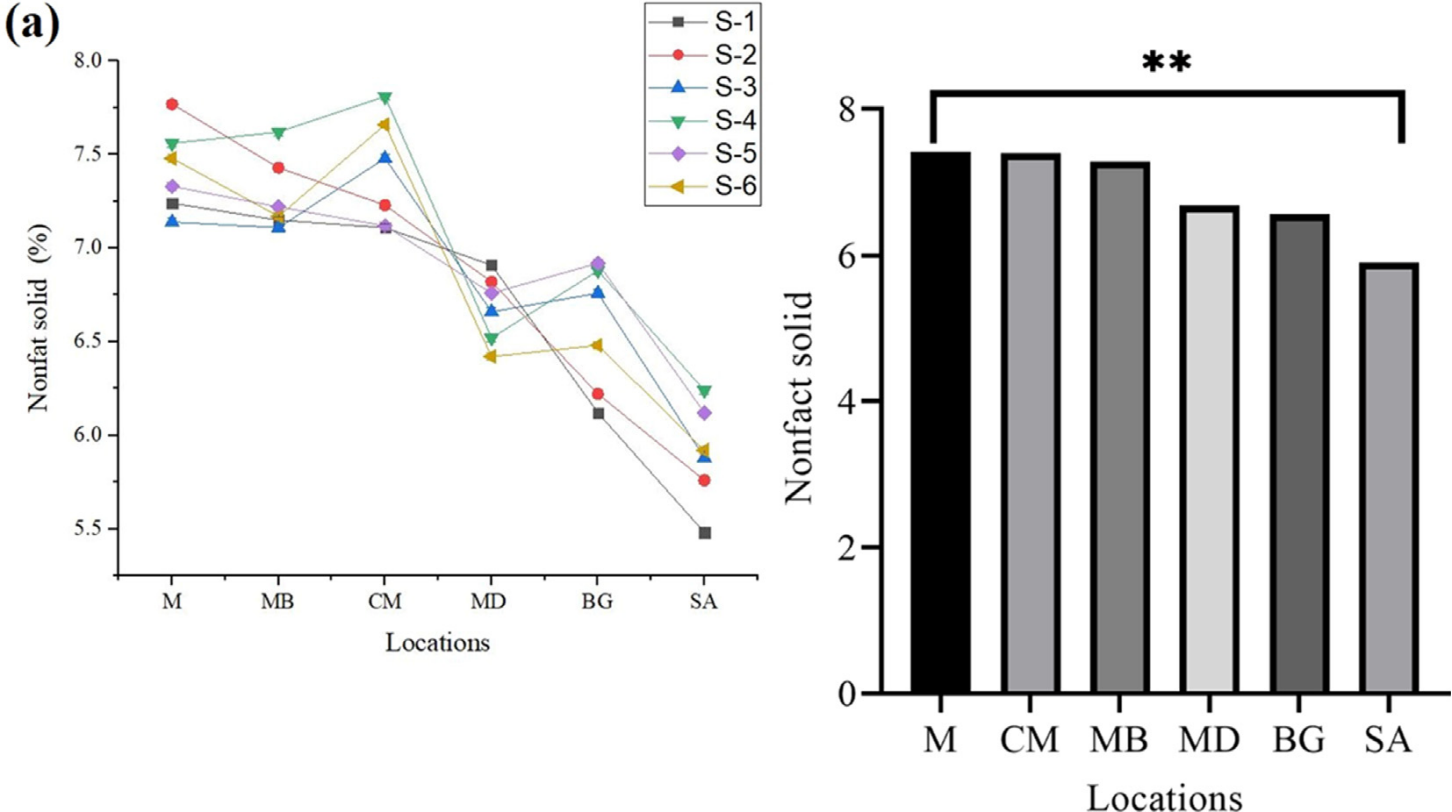
Presence of Nonfat solid in fresh milk obtained from various regions.

After the determination of SNF content, mean values were recorded as 6.68 (MD), 7.40 (CM), 5.90 (SA), 7.42 (M), 6.56 (BG) and 7.28 (MB) alternatively. Sample (M) showed the highest value of SNF as 7.42, followed by sample (CM) to be 7.40, and the minimum value of SNF content was noted to be 5.90 and 6.56 in sample SA and BG, alternatively. In conclusion, the Highest solid non-fat (%) was observed in M and the lowest in SA (Fig. 8a, b).

### 3.10 Chemical adulterants in samples of milk

#### 3.10.1 Formalin detection in fresh milk samples

The adulteration of Formalin was also negative in all the samples collected from the said area. Neutralizer detection in fresh milk samples: Generally, off-flavors and odors are covered with neutralizers in milk and harmonize Acidity and pH values. In the present study, sample MD was negative for formalin adulteration, while all other samples were positive… The Maximum percentage of Formalin was detected in CM at 50% whereas minimum was detected in in MB (20%) and SB (25%), followed by Bg (30%) and M (40%).

#### 3.10.2 Detergents, H_2_O_2,_ and starch detection in various samples of milk

The present study finding indicated that in all samples no detergent was detected. However, in all the milk samples collected from a different area showed negative Hydrogen peroxide, and starch (Table. 3).

**Table 3:** Adulteration percentage in sample of fresh milk collected from various regions.

Source of milk	Sample (n)	Urea	Formalin	Neutralizer	Detergents	H_2_O_2_	Starch
**MD**	6	0	0	0	0	0	0
**CM**	6	2(22%)	0	3(50%)	0	0	0
**SB**	6	0	0	2(25%)	0	0	0
**M**	6	0	0	3(40%)	0	0	0
**BG**	6	0	0	2(30%)	0	0	0
**MB**	6	3(30%)	0	2(20%)	0	0	0

## 4 Discussion

Milk has always been a vital cradle for obtaining primary nutrients for mammals. Dairy products like butter, cream, yogurt, sour milk, ghee, etc., are developed from milk sourced from buffaloes, goats, and cows. Quality nutrients like fats, proteins, carbohydrates, and minerals are received from milk in a ratio that any single food source cannot provide. Consumers always ask for milk and dairy products enriched in nutrients (Nicolaou et al., 2010). It is important to carry out a physicochemical analysis of milk by determining its chemical composition, physical attributes, and nutritional and microbial values (Czerniewicz et al., 2006). Adulterated foods can be detrimental to health because they may contain poisonous chemicals or a deficit of vital and basic nutrients essential for development and growth. Cheaper substances like cane sugar, lake water, and milk powder are normally added to milk to make them adulterated and maximize profit margin. Adulterating milk with water reduces SNF (solid, not fats) components, particularly protein, one of the essential nutrients required for normal growth and development. Therefore, the amount of SNF can determine the amount of water added to milk (Santos et al., 2013). The consequences of milk adulteration include a risk to consumers' safety, lack of quality in the finished product, and financial damages (Mabrook and Petty, 2003). If water is added with partial or complete milk skimming, financial losses will be even higher. The current study was designed to address the physico-chemical qualities of milk and its adulteration in the city of Mardan, Khyber Pakhtunkhwa (KP)-Pakistan, from the selected milk shops.

This study revealed lower fat, protein, and water content in the selected milk samples. Milk obtained from the local market with reduced-fat contents can be attributed to water adulteration. In some cases, skimming or withdrawing milk fats from such samples can also be the rationale behind this scenario. Weekes (2008) found lower fat content in collected milk samples from the local market. Moreover, the differences in the ratio of fats can also be implied to mismanagement of milk at the farm level, cattle breeds, and adulterants. Fat content in buffalo milk is normally about 7.45%. (Gantner et al., 2015) introduced a comparative summary of the total contents of milk and fats obtained from several species and human milk. Significant differences were noted in energy content, ash, protein, lactose, and fat content in the overall composition of milk from numerous milk species. Some attributes were also noted to be similar among non-ruminants and ruminants milk. Between non-ruminants and human milk, the fat globule membrane was identical, while this structure had considerable differences from ruminants. The size of fat globules was considerably different between species and highly correlated to the milk fat content, regardless of the specie. The milk protein content was different from animal to animal and breed (Joslyn and Heid, 2009). These differences might be due to the nutritional level and genotypic differences in cows or the addition of water.

The difference in water content may be attributed to the additional changes in water in the milk samples. The study revealed that milk quantity is adulterated with water, and in many cases, it is not safe for consumption. The addition of neutralizers in the milk can be attributed to the pH of milk samples. According to Bylind (2008), the normal pH of milk is 6.7. Neutralizers are added to increase milk's shelf life and stop curd formation (Afzal et al., 2011). The PH of milk increases with the addition of a neutralizer to milk, and milk turns into alkaline, and the PH value is frequently higher than 8.0 (Subrahmanyam, 2002). Ladokun and Oni (2014) studied various fermented milk foodstuffs made from various milk natures, which determined that the fresh milk samples were to some extent acidic, i.e., soymilk (6.4) goat milk (6.2), cow milk (6.3) and coconut milk (6.0). The PH outcomes of different fermented milk at 0 h of production were (5.24), cow milk (5.85), soymilk (5.73), and coconut milk (5.98). On the other hand, at 72 h, all the milk samples become more acidic because the fermentation and PH values become low.

The density of the fresh milk is subjected to the milk's contents and the temperature. The density can be assessed by summing the individual density of each content of milk, and the overall density depends upon the temperature of the milk. As milk is a complex mixture of multiple components, determining a single component through density only is not wise. Yet, this density measurement provides information about differences from standard milk composition in water adulteration.

Milk is a complex mixture of multiple components. It is a primary source for young mammals obtained from mammary glands (Ilie et al., 2010). In some parts of the globe, camels, sheep, and goats are among the main sources of milk and milk products, yet cow milk has its major place in dairy products worldwide, and its importance cannot be ignored. This is the reason why cow milk has been under detailed investigation in research (Forsbäck et al., 2010). The composition and quality of milk are affected by multiple factors, including the physical status of an animal, nutritional condition, and age. Generally, milk comprises water at 87–88% and total solids at 12–13%. Among the total solids, 9% are Solid, Not Fats (SNF), and the remaining 4% are fats. SNF includes lactose, proteins, vitamins, minerals, and other components (Pathot, 2019).

Milk salts are composed of sulfates, chlorides, phosphates, citrates, carbonates, and bicarbonates of calcium, magnesium, potassium, and sodium. Approximately twenty other elements are found in milk in a rare amount, including boron, iodine, iron, lead, zinc, copper, and manganese (Edwards et al., 2014). Sodium can be found in milk in a very minute quantity. About 120 mg of sodium are present in 250 ml milk (Bruckmaier et al. 2004). Electric conductivity is the electric current concentration that can pass through the milk. Different compounds have different conductivity levels. This level is enhanced by the presence of inorganic compounds and the number of salts in a sample. These ions constantly support to permit the electricity swiftly. Several other parameters also affect the conductivity. Temperature is one of the main factors; the higher the temperature of a sample, the higher the conductivity and vice versa (Hillerton and Semmens, 1999). The soluble salt content in milk essentially enhances the electrical conductivity in milk. Fats and lactose are not good conductors of electricity, decreasing the EC of milk. Peptides and proteins present in milk have the least effect on conductivity (Lee and Choudhary, 2006). At room temperature, milk obtained from healthy animals possesses 4–5.50 mS/cm electrical conductivity (Yoshida et al., 2005). Park et al. (2007), the electrical conductivity of milk obtained from a healthy cow at 37 °C is 4.54 mS/cm. This level reaches upto 6.31 mS/cm and even higher when the cow suffers mastitis, a declined udder health condition. The relationship between the somatic cells and the electrical conductivity of milk is about 0.91 (Nowak et al., 1990).

In milk, when SNF (solids, not fats) quantity increases, the sensory attributes of fats and cream reduce, and the characteristics of the odor of skimmed milk increase. That is why earlier detection of SNF quantity in milk can assist manufacturers in producing quality milk products and bring safety in terms of sugar and other types of adulteration (Barham et al., 2015). The percentage of milk fat can indicate absolute variation compared to other ingredients. There is a direct relationship between the number of fats in milk and solid not fats in the milk. If SNF is increased, the fat content will be decreased and vice versa (Park et al., 2007). SNF (Solid Not Fats) comprises numerous components, including carbohydrates (lactose in abundance), proteins (Casein and lactoalbumin mainly), and several minerals, including calcium and phosphorus). A sufficient quantity of Riboflavin is also present in milk, and some extra vitamins are soluble in water (Iqbal, 2017). Various interstate and federal organizations govern exports and imports of milk with minimum and maximum fats content and SNF specifications. Milk should have a minimum of 3.25% milk fats and 8.25% SNF (Clare et al., 2003).

The local market of Mardan, from which samples were collected, did not show signs of formalin adulteration. The current research findings are similar to Mansour et al., 2012, Lateef et al., 2009, where both studies concluded with a lack of formalin adulteration in their samples. Formalin is used for the preservation purpose of milk samples. Yet, it may have some adverse effects on health, including abdominal pain, vomiting, diarrhea, breathing issues, rise in temperature, blindness, and irregular and weaker pulse rate (Mcgwin et al., 2010).

## 5 Conclusion

Fats provide about half of the calories in milk. A high-quality protein called Casein is present in milk that contains almost all the amino acids essential for normal body growth and tissue regeneration. The difference in water content may be attributed to the additional changes in water in the milk samples. The addition of neutralizers in the milk can be attributed to the pH of milk samples. The density of the fresh milk is subjected to the milk's contents and the temperature. Approximately twenty elements are found in milk in the rare amount, including boron, iodine, iron, lead, zinc, copper, and manganese. Several parameters affect the conductivity of milk. Temperature is one of the main factors; the higher the temperature of a sample. The percentage of milk fat can indicate absolute variation compared to other ingredients. The local market of Mardan collected samples, and neither showed signs of formalin adulteration nor neutralizer adulteration. Even hydrogen peroxide adulteration, starch, and detergent adulteration were not found in district Mardan samples. Therefore, the Fortification of milk with micronutrients is recommended, which is essential for every individual. However, further research should be designed to blend different milk lots available in the local market to investigate their contents.

## Declaration of Competing Interest

The authors declare that they have no known competing financial interests or personal relationships that could have appeared to influence the work reported in this paper.
